# Hypothermic cardiac arrest far away from the center providing rewarming with extracorporeal circulation

**DOI:** 10.1186/1865-1380-5-7

**Published:** 2012-02-01

**Authors:** Eckhard Mark, Olaf Jacobsen, Astrid Kjerstad, Torvind Naesheim, Rolf Busund, Ramez Bahar, Jon Kjetil Jensen, Per Kristian Skorpen, Lars J Bjertnaes

**Affiliations:** 1 Department of Emergency Medical Services, University Hospital of North Norway, Trom-sø, Norway; 2 Department of Anesthesiology, University Hospital of North Norway, Tromsø, Norway; 3 Department of Thoracic and Cardiovascular Surgery, University Hospital of North Norway, Tromsø, Norway; 4 Department of Medical Engineering, University Hospital of North Norway, Tromsø, Norway; 5 Department of Medicine, Stokmarknes Hospital, Stokmarknes, Norway; 6 Anesthesia and Critical Care Research group, Institute of Clinical Medicine, Faculty of Health Sciences, University of Tromsø, Tromsø, Norway

## Abstract

A 41-year-old man suffered hypothermic cardiac arrest after water immersion and was transported to our university hospital by ambulance helicopter for rewarming on cardiopulmonary bypass. He resumed spontaneous cardiac activity 6 h 52 min after cardiac arrest and recovered completely.

## Background

In the Northern hemisphere, most victims of accidental hypothermic cardiac arrest have a history of drowning or entombment by avalanches. Consequently, asphyxia and cardiac arrest develop before the body temperature has fallen to a sufficiently low level for oxygen demand to meet the supply. However, some patients have the advantage that they can breathe while being cooled until the heart stops [1-5].

## Case report

An alcohol-intoxicated man, 41 years of age, fell into a river on the coast of North Norway after leaving a party between 3 and 4 o'clock a.m. on 30 December. Approximately 1 h later, passers by spotted him shouting for help and pulled him out of the water. One of them undressed and attempted to warm him by skin-to-skin contact at the prevailing air temperature of -2°C. The patient lost consciousness and stopped breathing as the paramedics lifted him into the ambulance at 04.45 a.m. One of them started cardiopulmonary resuscitation (CPR) and continued it during the drive to the local hospital. Upon arrival at 05.01 a.m., his electro-cardiogram (ECG) was isoelectric, rectal temperature was 27.5°C, and arterial blood gases displayed pH 7.00, PaCO_2 _10.60 kPa, PaO_2 _3.60 kPa, HCO_3 _^-^18.5 mmol/l, and BE -11.3 mmol/l. He had no visible injuries except for a wound in the occipital region. He was endotracheally intubated and received 90 mmol of trimetamol (Tribonat^®^, Fresenius Kabi AS, Oslo, Norway) intravenously (IV). Following two IV injections of 1 mg epinephrine (Adrenalin^®^, Nycomed Pharma AS, Asker, Norway), his ECG shifted to ventricular fibrillation (VF), but attempts at defibrillation failed. At 6.00 o'clock, the physician in charge contacted the ambulance dispatch center of the University Hospital of North Norway (UNN) in Tromsø (latitude: 69°, 40' North). The anesthesiologist on duty for the helicopter and the thoracic surgical team decided to fly the 260 km (140 NM) to the local hospital and bring the patient to UNN for rewarming by means of extracorporeal circulation. The helicopter that was prepared for instrument flights took off at 06.45 a.m. When starting the return flight from the local hospital at 08.00 a.m., the stretcher was turned across the cabin. This facilitated optimum access for manual ventilation (10 inflations per min, FiO_2 _1.0) and external cardiac compression (100 compressions per min) carried out alternately by the anesthesiologist and a nurse anesthetist with no hands off time [6].

After landing at UNN at 08.45 a.m., the patient was transferred to the emergency room where his arterial blood gases showed: pH 6.88, PaO_2 _26.20 kPa, PaCO_2 _7.62 kPa, and base excess -21 mmol/l. Serum creatine phosphokinase (CPK) and myoglobin were 14,000 μg/l and 3,017 U/l, respectively, K^+ ^5.9 mmol/l, Ca^2+ ^1.7 mmol/l, and rectal temperature 25°C. The ECG displayed a coarse VF. The occipital wound was sutured, and at 09.18 a.m., he was connected to a cardiopulmonary bypass (CPB) using a fully heparin-coated system with access via the right femoral vein and artery. After 30 min on CPB, 5 h 3 min after cardiac arrest, his esophageal temperature had increased to 33°C, and the VF was electro-converted into a nodal rhythm, which resulted in asynchronous ventricular contractions, as assessed by transesophageal echocardiography. After 2 h 21 min on CPB, his rectal temperature had risen further to 36.5°C paralleled by gradually improving cardiac function. He was weaned off CPB at 11.37 a.m., 6 h 52 min after cardiac arrest, maintaining a mean systemic arterial pressure of 55 mmHg supported by infusions of norepinephrine (Noradrenalin^®^, NAF, Oslo, Norway) 130 ng/kg/min, pitressin (Glypressin^®^, Ferring legemidler, Oslo, Norway) 120 ng/kg/min, and milrinon (Corotrop^®^, Sanofi-Aventis Norge, Oslo, Norway) 120 ng/kg/min. At 12.30 a.m., he was transferred to the ICU. Then, his serum myoglobin was 17,000 μg/l, ASAT 1,242 U/l, ALAT 214 U/l, creatinine 195 μmol/l, CK-MB 61 μg/l, thrombocyte count 50 × 10^9^/l, fibrinogen 1.0 g/l, D-dimer 10.7 μg/l FEU, and antithrombin III 27%, and the ethanol gel test was positive. Concomitantly, arterial oxygen saturation (SaO_2_) reached a nadir of 73%. He was subjected to airway recruitment, and the SaO_2 _level rose to 90%.

Postoperatively, his condition was complicated with pneumonia requiring antibiotics and continuation of pressure-controlled mechanical ventilation (Servo I, MAQUET GmbH & Co., Rastatt, Germany). Moreover, renal failure as assessed by hourly diuresis of below 20 ml/h; serum urea and creatinine levels of 11.3 mmol/l and 195 μmol/l, respectively, indicated the need for veno-venous hemofiltration (Prismaflex system, Gambro, Lund, Sweden). The coagulation dysfunction normalized after transfusion of freshly frozen plasma and thrombocyte concentrates, and the patient's liver function regressed during the first week. He was weaned off mechanical ventilation and extubated on 3 January, and was transferred to the local hospital 12 days later. In a letter 3 years after the accident he wrote that he has quit smoking and abusing alcohol, and works as a logistic consultant. He has no neurological sequelae and enjoys life with his family. He wanted us to emphasize in the case report that the accident became an incentive for him to change to a healthier lifestyle.

## Discussion

We are not aware of previous reports of successful resuscitation after hypothermic cardiac arrest of a comparably long duration. A PubMed search revealed the case of an alcohol-intoxicated man who was found outdoors with cardiac arrest and a rectal temperature of 23.2°C. Subjected to surface rewarming combined with intraperitoneal lavage and intravenously infused warm fluids at the local hospital, he resumed spontaneous circulation with sinus rhythm at a rectal temperature of 28°C 6 h after CPR was started. However, neurological examination before discharge revealed "slow cerebration" and peripheral neurological dysfunction in both arms [3].

Walpoth and co-workers surveyed the outcomes of victims of accidental hypothermic cardiac arrest varying between 30 min and 4 h who were successfully rewarmed with extracorporeal circulation [4]. Recently, colleagues at our hospital reported the case of a female off-piste skier who fell and was trapped head down between rocks and overlying ice, and sprinkled with glacial water in her face until cardiac arrest. With a body temperature reaching a nadir of 13.7°C and cardiac arrest for almost 3 h, she was successfully resuscitated on CPB followed by a period of extracorporeal membrane oxygenation because of severe respiratory failure. She had no sequelae 2 years later [5].

Regarding our patient, a well-equipped ambulance dispatch center and its staff of specialized nurses and flight coordinators, supervised by the anesthesiologist on duty for the helicopter, played an important role by coordinating the total pre- and intra-hospital resources. Because of other similar actions during the last few years, the center, which is an integral part of our Department of Emergency Medical Services, has obtained experience in coordinating the transfer of patients with cardiac arrest with rotor or fixed-wing aircraft over long distances [5]. Our university hospital is the only center for cardiovascular surgery in the area providing rewarming with extracorporeal circulation. The next center is located in Trondheim, approximately 600 km to the south. Although the dispatch center could have chosen to use a correspondingly staffed and equipped ambulance plane, flying at double speed, the helicopter was preferred because it could provide "door-to-door" transfer.

Arriving at the local hospital under ongoing CPR, the rectal temperature of the patient already had fallen by 10°C, which corresponds to a 60-70% decrease in cerebral metabolic rate, based on a 6-7% reduction per degree fall in body temperature [7]. Presumably, his alcohol intoxication helped him avoid most of the shivering and the resulting increase in oxygen consumption that normally occurs during exposure to cold water. In anesthetized dogs cooled from 37 to 25°C core temperature, investigators noticed that cardiac output fell spontaneously by 65% [8]. Although performed by experienced specialists according to the guidelines [6], we have no evidence of the efficacy of the cardiac compressions. However, by extrapolating from the results of the latter investigators, we assume that a cardiac output of 30-40% of normal should be sufficient to balance the oxygen demand at a body temperature below 27°C. The latter estimate also corresponds to a decrease in cerebral blood flow to 40% of normal, which was observed in pigs undergoing external manual cardiac compression during normothermic circulatory arrest [9]. Although uninterrupted cardiac compression from the scene of the accident might have satisfied most of the patient's oxygen demand until he was connected to CPB, acidosis made likely that he had suffered some degree of hypoxia. Moreover, the fact that relatively high doses of norepinephrine, pitressin, and milrinon were necessary to wean him off CPB, even with a low systemic arterial pressure, suggests that he was subjected to "rewarming shock." It is believed that this type of circulatory failure, which has been observed in various species after hypothermia and rewarming, and often with a fatal outcome, is associated with intracellular Ca^2+ ^overload [10]. However, the exact mechanism of this dysfunction of intracellular ion transport remains unsettled.

High serum levels of myoglobin and CPK indicate that the patient had a rhabdomyolysis that, most likely, triggered his renal and hepatic failures in concert with disseminated intravascular coagulation [11]. Thus, including a transient central nervous impairment and respiratory failure, our patient suffered at least five organ failures. However, in addition to a complete physical and mental recovery, he has also adopted a healthier lifestyle.

## Conclusion

To the best of our knowledge, 6 h 52 min is the longest time reported until the return of spon-taneous circulation and recovery without physical or mental sequelae after hypothermic cardiac arrest. We believe that well-integrated pre-hospital as well as local and university hospital medical services were important prerequisites for the successful outcome of this patient.

## Abbreviations

CPB: cardiopulmonary bypass; CPR: cardiopulmonary resuscitation; ECG: electrocardiogram; IV: intravenously; UNN: University Hospital of North Norway; VF: ventricular fibrillation

## Consent

Written informed consent was obtained from the patient for publication of this case report. A copy of the written consent is available for review by the Editor-in-Chief of this journal.

## Competing interests

The authors declare that they have no competing interests.

## Authors' contributions

EM was the anesthesiologist on board the helicopter, conceived the idea for and drafted the case report. OJ was the nurse anaesthetist on board the helicopter, AK and TN were cardiac anaesthesiologists on duty, RB and RB were thoracic surgeons, JKJ was the perfusionist, and PKS was in charge of treatment at the local hospital. LJB wrote and formatted the manuscript. All the authors have read and approved the final manuscript.

## References

[B1] Bierens JJ van der Velde EA van Berkel M van Zanten JJ Submersion in The Netherlands: prognostic indicators and results of resuscitation Ann Emerg Med 1990 19 1390 5 10.1016/S0196-0644(05)82604-6 2240751

[B2] Boyd J Brugger H Shuster M Prognostic factors in avalanche resuscitation: a systematic review Resuscitation 2010 81 645 52 Review 10.1016/j.resuscitation.2010.01.037 20371145

[B3] Lexow K Severe accidental hypothermia: survival after 6 hours 30 minutes of cardiopulmonary resuscitation Arctic Med Res 1991 50 Suppl 6 112 4 1811563

[B4] Walpoth BH Walpoth-Aslan BN Mattle HP Radanov BP Schroth G Schaeffler L Fischer AP von Segesser L Althaus U Outcome of survivors of accidental deep hypothermia and circulatory arrest treated with extracorporeal blood warming N Engl J Med 1997 337 1500 5 10.1056/NEJM199711203372103 9366581

[B5] Gilbert M Busund R Skagseth A Nilsen PA Solbø JP Resuscitation from accidental hypothermia of 13.7 degrees C with circulatory arrest Lancet 2000 355 375 6 10.1016/S0140-6736(00)01021-7 10665559

[B6] https://www.erc.edu/

[B7] Michenfelder JD Anesthesia and the Brain: Clinical, Functional, Metabolic, and Vascular Correlates 1988 New York: Churchill Livingstone

[B8] Tveita T Mortensen E Hevrøy O Refsum H Ytrehus K Experimental hypothermia: effects of core cooling and rewarming on hemodynamics, coronary blood flow, and myocardial metabolism in dogs Anesth Analg 1994 79 212 8 7639353 10.1213/00000539-199408000-00002

[B9] Rubertsson S Karlsten R Increased cortical cerebral blood flow with LUCAS; a new device for mechanical chest compressions compared to standard external compressions during experimental cardiopulmonary resuscitation Resuscitation 2005 65 357 63 10.1016/j.resuscitation.2004.12.006 15919574

[B10] Kondratiev TV Wold RM Aasum E Tveita T Myocardial mechanical dysfunction and calcium overload following rewarming from experimental hypothermia in vivo Cryobiology 2008 56 15 21 10.1016/j.cryobiol.2007.09.005 17983615

[B11] Huerta-Alardín AL Varon J Marik PE "Bench-to-bedside review: rhabdomyolysis - an overview for clinicians" Critical Care 2005 9 158 69 doi:10.1186/cc2978 10.1186/cc3221 15774072 PMC1175909

